# Cerebral Blood Volume Calculated by Dynamic Susceptibility Contrast-Enhanced Perfusion MR Imaging: Preliminary Correlation Study with Glioblastoma Genetic Profiles

**DOI:** 10.1371/journal.pone.0071704

**Published:** 2013-08-19

**Authors:** Inseon Ryoo, Seung Hong Choi, Ji-Hoon Kim, Chul-Ho Sohn, Soo Chin Kim, Hwa Seon Shin, Jeong A. Yeom, Seung Chai Jung, A. Leum Lee, Tae Jin Yun, Chul-Kee Park, Sung-Hye Park

**Affiliations:** 1 Department of Radiology, Seoul National University College of Medicine, Seoul, Korea; 2 Department of Neurosurgery, Seoul National University College of Medicine, Seoul, Korea; 3 Department of Pathology, Seoul National University College of Medicine, Seoul, Korea; City of Hope, United States of America

## Abstract

**Purpose:**

To evaluate the usefulness of dynamic susceptibility contrast (DSC) enhanced perfusion MR imaging in predicting major genetic alterations in glioblastomas.

**Materials and Methods:**

Twenty-five patients (M:F = 13∶12, mean age: 52.1±15.2 years) with pathologically proven glioblastoma who underwent DSC MR imaging before surgery were included. On DSC MR imaging, the normalized relative tumor blood volume (nTBV) of the enhancing solid portion of each tumor was calculated by using dedicated software (Nordic TumorEX, NordicNeuroLab, Bergen, Norway) that enabled semi-automatic segmentation for each tumor. Five major glioblastoma genetic alterations (epidermal growth factor receptor (EGFR), phosphatase and tensin homologue (PTEN), Ki-67, O6-methylguanine-DNA methyltransferase (MGMT) and p53) were confirmed by immunohistochemistry and analyzed for correlation with the nTBV of each tumor. Statistical analysis was performed using the unpaired Student t test, ROC (receiver operating characteristic) curve analysis and Pearson correlation analysis.

**Results:**

The nTBVs of the MGMT methylation-negative group (mean 9.5±7.5) were significantly higher than those of the MGMT methylation-positive group (mean 5.4±1.8) (p = .046). In the analysis of EGFR expression-positive group, the nTBVs of the subgroup with loss of PTEN gene expression (mean: 10.3±8.1) were also significantly higher than those of the subgroup without loss of PTEN gene expression (mean: 5.6±2.3) (p = .046). Ki-67 labeling index indicated significant positive correlation with the nTBV of the tumor (p = .01).

**Conclusion:**

We found that glioblastomas with aggressive genetic alterations tended to have a high nTBV in the present study. Thus, we believe that DSC-enhanced perfusion MR imaging could be helpful in predicting genetic alterations that are crucial in predicting the prognosis of and selecting tailored treatment for glioblastoma patients.

## Introduction

Malignant gliomas are the most common and lethal cancers originating in the brain. Glioblastoma multiforme (GBM, World Health Organization [WHO] Grade IV), the most aggressive subtype of glioma, has a dismal prognosis. Nevertheless, survival for patients with GBM has improved from an average of 10 months to 14 months after diagnosis in the last several years due to the development of various treatment options [1].

Improvement in treatment strategies has largely been based on the substantial progress in the identification of genetic alterations or profiles in GBMs, which enables tailored therapy. Inarguably, the most accurate method for the identification of genetic alterations in GBM is pathologic analysis. Nevertheless, the procedure for obtaining the brain tumor tissue for pathologic analysis is time consuming, costly, physician intensive and not always feasible, given its innate invasiveness [2], [3]. For example, it is potentially dangerous to sample tissue from patients who are in poor general condition for brain surgery, who have tumors in a critical portion of the brain or who have recurrent tumors on follow-up images after treatment involving surgery. These tissue-based methods are also often associated with sampling errors due to improper resection or biopsy of tumor tissues and heterogeneity of tumors, resulting in some cases with false genetic profile assignation. Moreover, preoperative insight into the genetic composition of the tumor is sometimes, if not often, necessary to guide preoperative chemotherapies to shrink the tumor.

Due to the need for less- or non-invasive means of predicting GBM genetic alterations, along with the recent tremendous advances in imaging techniques, there have been many attempts to use the imaging features of GBM with conventional MR and perfusion-weighted imaging (PWI) techniques as noninvasive radiophenotypic surrogates for genetic alterations [4]–[6]. However, similar attempts to correlate tumor blood volume (TBV) from dynamic susceptibility contrast (DSC) enhanced perfusion MR imaging with various genetic alterations in GBMs have not yet been conducted, except for a very recent study correlating TBV with epidermal growth factor receptor variant III (EGFRvIII) expression status in GBMs [3].

Our hypothesis is that GBMs with genetic profiles associated with poor prognosis would show high TBV values on DSC-enhanced perfusion MR imaging, because it is well known that the TBV value can predict the progression of GBMs [7]. Thus, the purpose of our study is to evaluate the usefulness of the TBV value from DSC-enhanced perfusion MR imaging in predicting major genetic alterations in glioblastomas.

## Materials and Methods

### Ethics Statement

This retrospective study was approved by the institutional review board of Seoul National University Hospital, and informed consent was waived.

### Patient Population

Using a computerized search of our hospital’s medical records and pathology files from November 2009 to February 2012, we identified 102 patients with pathologically proven GBM (World Health Organization [WHO] Grade IV) who had undergone surgery in our hospital. Sixty-one patients without preoperative DSC-enhanced perfusion MR imaging performed with a 3-tesla MR machine were excluded. An additional two patients, one with no immunohistochemistry (IHC) results and another with severe paramagnetic artifacts on MR imaging, were also excluded.

Among the remaining 39 patients, 10 patients had GBMs with an oligodendroglial component and four patients had a 1p 19q deletion, which are known to be associated with a more favorable prognosis than in those patients having general GBMs [8]–[13].

**Figure 1:**
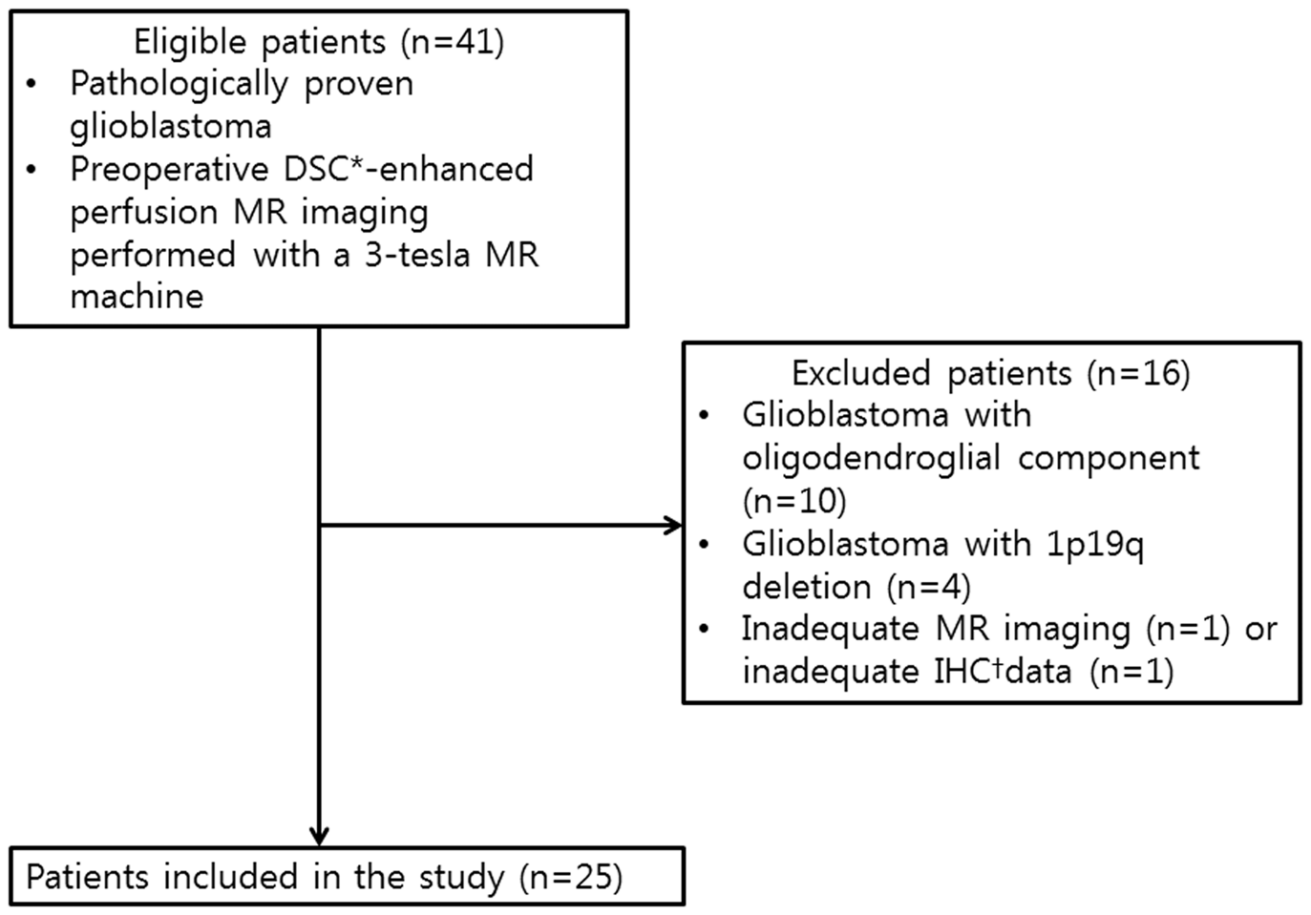
Flow diagram of patient selection and inclusion and exclusion criteria. Note– *DSC: dynamic susceptibility contrast, ^†^IHC:immunohistochemistry.

Finally, a total of 25 patients were included in our retrospective study (M:F = 13∶12, mean age: 52.1±15.2, age range: 26–71 years) (Figure 1). All examinations were performed within 15 days before the surgery (mean: 4.52±3.97 days, range: 1–15 days).

### MR Imaging Technique

All 25 patients underwent conventional MR imaging and DSC- enhanced perfusion MR imaging using a 3 T-scanner (Verio; Siemens Healthcare Sector, Erlangen, Germany) with a 32-channel head coil. The conventional MR imaging included T1-weighted imaging such as transverse spin-echo imaging before and after contrast enhancement, multi-planar reconstructed transverse and coronal imaging from a sagittal three-dimensional magnetization prepared rapid acquisition gradient echo (3D-MPRAGE) sequence before and after contrast enhancement and transverse T2-weighted turbo spin-echo sequences. Contrast-enhanced T1-weighted imaging was acquired after the intravenous administration of gadobutrol (Gadovist®, Bayer Schering Pharma, Berlin, Germany) with 0.1 mmol per kilogram (mmol/kg) of body weight. Transverse spin-echo T1-weighted images were obtained with the following parameters: repetition time (TR), 558 ms; echo time (TE), 9.8 ms; flip angle (FA), 70°; matrix, 384×187; field-of-view (FOV), 175×220 mm; section thickness, 5 mm; and number of excitations (NEX), 1. We obtained 3D-MPRAGE sequences using the following parameters: TR, 1500 ms; TE, 1.9 ms; FA, 9°; matrix, 256×232; FOV, 220×250 mm; section thickness, 1 mm; and NEX, 1. The parameters of transverse T2-weighted images were as follows: TR, 5160 ms; TE, 91 ms; FA, 124–130°; matrix, 640×510–580; FOV, 175–199×220 mm; section thickness, 5 mm; and NEX, 3.

The transverse DSC-enhanced perfusion MR imaging was obtained with a single-shot gradient-echo echo-planar sequences during the intravenous administration of gadobutrol with 0.1 mmol/kg of body weight at a rate of 4 mL/sec by use of a power injector (Spectris; Medrad, Pittsburgh, PA). A 30-mL bolus injection of saline was followed at the same injection rate. For each section, 60 images were acquired at intervals equal to the TR. The parameters were as follows: TR, 1500 ms; TE, 30 ms; FA, 90°; matrix, 128×128; section thickness, 5–6 mm; intersection gap, 1 mm; FOV, 240×240 mm; sections, 15–20; voxel size, 1.875×1.875×5 mm^3^; pixel bandwidth, 1563 Hz; and total acquisition time, 1 minute 30 seconds.

### Image Postprocessing and Perfusion Data Analysis

**Figure 2:**
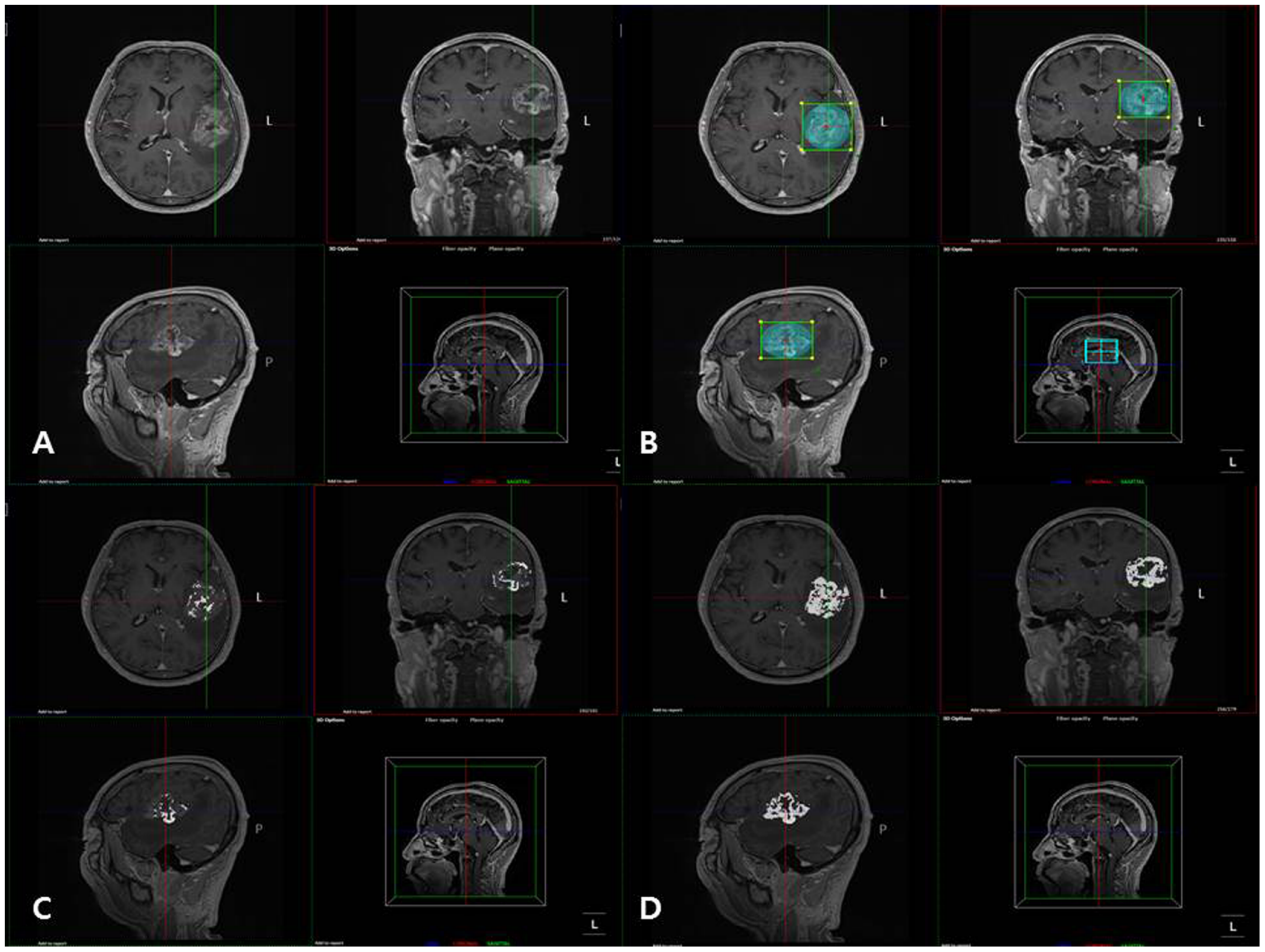
Clustering method was used as a semi-automatic segmentation of glioblastoma. (A) Contrast-enhanced T1-weighted images were used as structural images. (B) Volume of interest (VOI) was determined by a neuroradiologist. (C, D) VOIs were divided into seven tissue classes according to pixel values and class 6 and 7 were selected as enhancing tumor tissue. (C: class 6, D: class 6 and 7 together).

The DSC-enhanced perfusion MR images were processed by use of an MR perfusion analysis method including semi-automatic segmentation (Nordic TumorEx, NordicNeuroLab, Bergen, Norway), in which contrast-enhanced T1-weighted images (CE-T1WI) were used as structural images. There are three algorithms used for tissue segmentation in this program. (Threshholding, Seed growing and Clustering). Clustering was used in this study. The volume of interests (VOIs) determined by a neuroradiologist with 7 year-experience in neuroradiology based on structural images (i.e., CE-T1 WI) were divided into seven tissue classes. This clustering or classification is performed using an Expectation-Maximization algorithm. Pixels are assigned to the class with the highest probability of finding the vector whose elements are the pixel values in that class. Class 6 and 7 among the seven classes were chosen as tumor tissue in this study excluding nontumorous portions such as tissues associated with necrosis and edema (Figure 2). The relative cerebral blood volume (rCBV) maps were generated by use of established tracer kinetic models applied to the first-pass data [14], [15]. To reduce the recirculation effects, the ΔR2*(1/T2*) curves were fitted to a gamma-variate function, which is an approximation of the first-pass response as it would appear in the absence of recirculation or leakage. The dynamic curves were mathematically corrected to reduce contrast-agent leakage effects [16]. Normalization of rCBV maps is automatically performed using the mean value of the blood volume values outside the tumor without any intervention of observers. The normalized rCBV (nCBV) maps were presented as color overlays on structural images. The coregistration between the structural images and nCBV maps was performed based on geometric information stored in the respective data sets, automatically [17], [18]. However, manual correction was also performed before final coregistration to minimize the misresgistration caused by geometric distortions on echo-planar sequences. Finally, the normalized relative tumor blood volume (nTBV ) values of the tissue classified as tumor tissue (i.e., class 6 and 7 in this study) were calculated based on coregistered nCBV maps.

### Genetic Profile Analysis

In IHC studies of GBM, five genetic alterations, previously documented as important genetic markers for the grading of malignancy in gliomas, were identified [4], [6], [19], [20]. Among the genetic alterations, there were two proliferation markers (epidermal growth factor receptor [EGFR] and Ki-67), one DNA repair gene (O6-methylguanine-DNA methyltransferase [MGMT]) and two tumor-suppressor genes (phosphatase and tensin homologue [PTEN] and p53).

Each case was evaluated to estimate the positivity of neoplastic cells, according to the characteristics of each biomarker. The expression status of EGFR protein was visually scored using a three-tiered scale (1+, 2+, 3+) and a medium-power field (x200). To evaluate the expression status of p53 and the labeling index of Ki-67, an Aperio image analyzer (Aperio Technologies, Vista, CA) was utilized to calculate the percentage of positive tumor cells. For MGMT, its promoter methylation status was investigated using a methylation-specific polymerase chain reaction (MS-PCR) technique. The MGMT promoter-methylation status of a tumor did not always correspond to its MGMT immunohistochemical expression status (MGMT-loss or MGMT-no loss groups). IHC staining for PTEN was also described in terms of loss (PTEN-loss or PTEN-no loss group). Finally, the pattern of the genetic alterations was analyzed for correlation with nTBV values as determined by DSC-enhanced perfusion MR imaging.

### Statistical Analysis

All statistical analyses were performed using a statistical software program (MedCalc, version 11.1.1.0; MedCalc, Mariakerke, Belgium). To evaluate the correlation between EGFR expression status and nTBV values and the correlation between PTEN expression status and nTBV, an ANOVA test and an unpaired Student t test were used, respectively. In the EGFR expression-positive subgroup analysis, the unpaired Student t test and receiver operating characteristic (ROC) curve analysis were applied to correlate nTBV parameters and PTEN expression status. The unpaired Student t test and ROC curve analysis were also used to determine whether the nTBV values of the MGMT methylation-positive group significantly differed from those of the MGMT methylation-negative group. In the ROC analysis, cutoff values that provided a balance between sensitivity and specificity for prediction of the genetic profiles were determined by selecting the coordinate that was nearest the left upper corner (ie, [0,1 ]) on each ROC curve, and values higher than the cutoff values were considered to indicate loss of PTEN expression or nonmethylation of MGMT promoter [21].

The diagnostic accuracies based on the nTBV values were estimated by calculating the area under the ROC curves (Az). The sensitivities and specificities at these cutoff levels were calculated, along with 95% confidence intervals (CIs).

Pearson’s correlation analysis was performed to measure the significance of the association between the nTBV parameters and two genetic alterations, specifically, Ki-67 labeling index and p53 expression status.

A multivariable stepwise logistic regression model was used to determine which genetic alteration is most strongly associated with nTBV value.

Leave-one-out cross-validation (LOOCV) test was also done to evaluate the accuracy of nTBV value in predicting the genetic profiles.

The data for each parameter were assessed for normality with the Kolmogorov-Smirnov test. In all tests, *P* values less than.05 were considered statistically significant.

## Results

**Figure 3:**
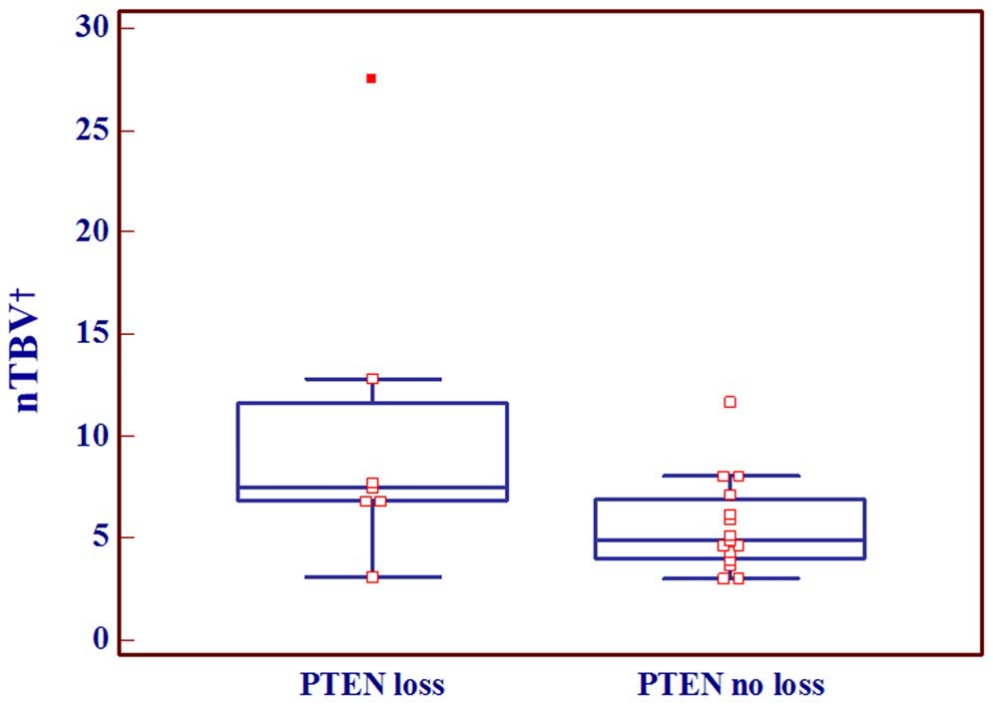
Box and whisker diagram displaying the relationship between normalized relative tumor blood volume (nTBV) and PTEN expression status in EGFR expression-positive group. The EGFR expression-positive glioblastomas with PTEN loss showed statistically higher nTBV values than those without PTEN loss. Note– ^†^nTBV: normalized relative tumor blood volume.

There were no significant differences in nTBV values among the groups with different EGFR expression statuses (p = .667). In terms of the loss of PTEN, we found no significant difference between the groups with the loss of PTEN (mean: 9.3±8.0, n = 8) and without the loss of PTEN (mean: 5.8±2.2, n = 17), (p = .09). However, in subgroup analysis with the EGFR expression-positive group (n = 22), the nTBV values of the subgroup (n = 7) with loss of PTEN gene expression (mean: 10.3±8.1) were significantly higher than those of the subgroup (n = 15) without loss of PTEN gene expression (mean: 5.6±2.3) (p = .046). The nTBV values according to the EGFR expression and PTEN expression statuses along with the results of Kolmogorov-Smirnov test are summarized in table 1 and figure 3. In the ROC curve analysis of EGFR expression-positive group, the Az value of nTBV for predicting PTEN loss was 0.733. The cutoff value that provided a balance between sensitivity and specificity was 8.05 and the corresponding sensitivity and specificity were 100% and 44.4%, respectively (table 2).

**Table 1:** nTBV values according to genetic alterations.

Genetic alteration	nTBV*	p-value†	KS test‡
**EGFR expression(+) group (n = 22)**			
PTEN loss (n = 7)	10.3±8.1	0.046	Accepted(0.602)
PTEN no loss (n = 15)	5.6±2.3		Accepted(0.319)
**MGMT methylation status (n = 25)**			
Methylation (+) (n = 16)	5.4±1.8	0.046	Accepted(0.845)
Methylation (−) (n = 9)	9.5±7.5		Accepted(0.594)

Note − Unless otherwise specified, the data are the means ± standard deviations.

* nTBV: normalized relative tumor blood volume.

† P-value for the comparison of means was calculated using unpaired Student’s t-test,

‡ KS test: Kolomogorov-Smirnov test for distribution normality.

**Table 2:** Az values, sensitivities, and specificities of nTBV in predicting PTEN loss or MGMT methylation status of GBM.

	PTEN loss		MGMT promoter nonmethylation	
	Value	95% CI	Value	95% CI
Az	0.674	0.459, 0.846	0.733	0.504, 0.896
Sensitivity (%)*	100	79.4, 100	73.3	44.9, 78.8
Specificity (%)*	44.4	13.7, 78.8	85.7	42.2, 97.6

Az, area under the ROC curve; CI, confidence interval.

* The cutoff values that provided a balance between sensitivity and specificity for predicting the genetic profiles were 8.05 for PTEN loss, and 6.19 for MGMT promoter nonmethylation.

**Figure 4:**
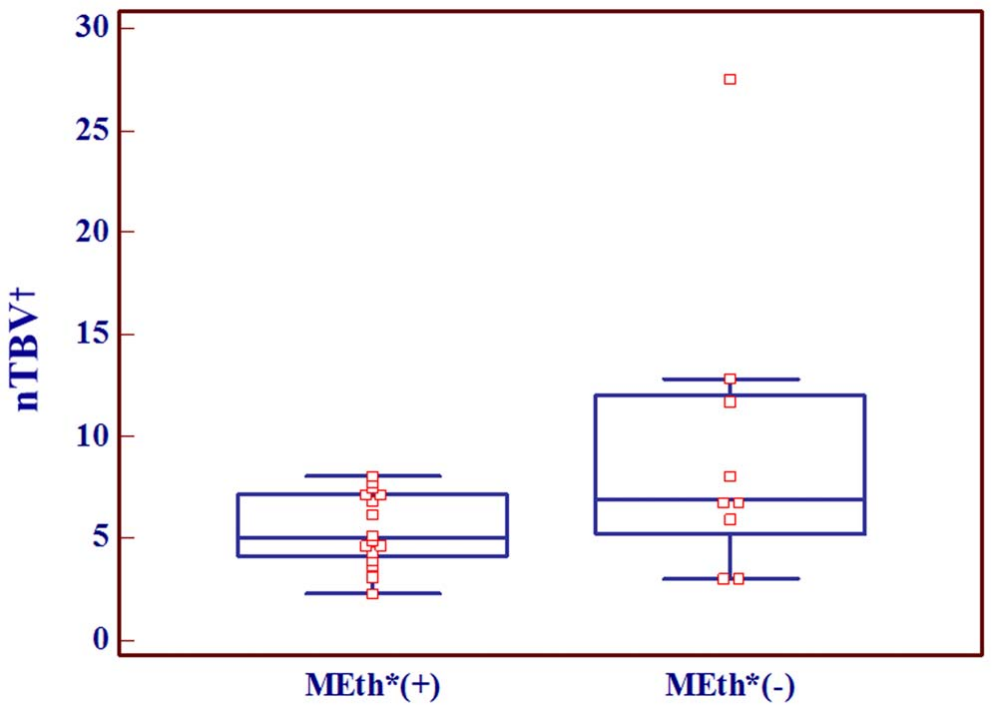
Box and whisker diagram displaying the relationship between normalized relative tumor blood volume (nTBV) and MGMT promoter methylation status. The MGMT methylation-positive group showed statistically lower nTBV values than the MGMT methylation-negative group. Note– *MEth: MGMT promoter methylation, ^†^nTBV: normalized relative tumor blood volume.

The nTBV values were significantly lower in the MGMT methylation-positive group (mean:5.4±1.8, n = 16)) than those in the MGMT methylation-negative group (mean: 9.5±7.5, n = 9) (p = .046) (table 1 and figure 4). In the ROC curve analysis, Az of nTBV for predicting MGMT methylation was 0.677. The sensitivity and specificity at the cutoff value of 6.19 were 73.3% and 85.7%, respectively (table 2).

With LOOCV test, the accuracies of nTBV in predicting PTEN loss in EGFR positive group and MGMT promoter methylation were 72.7% (16/22) and 76.0% (19/25), respectively.

**Figure 5:**
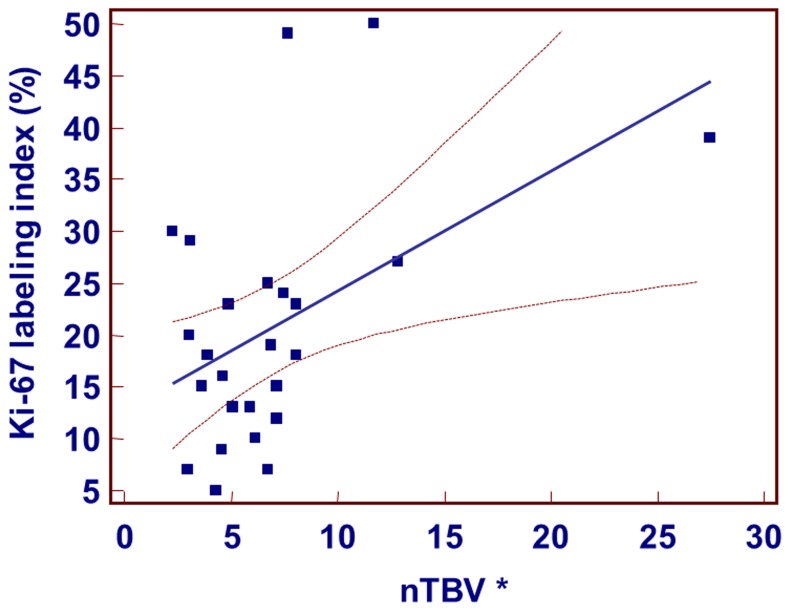
Scatter diagram and regression line display the relationship between nTBV and Ki-67 labeling index. There was strong positive correlation between the Ki-67 labeling index and nTBV. Note– *nTBV: normalized relative tumor blood volume.

**Figure 6:**
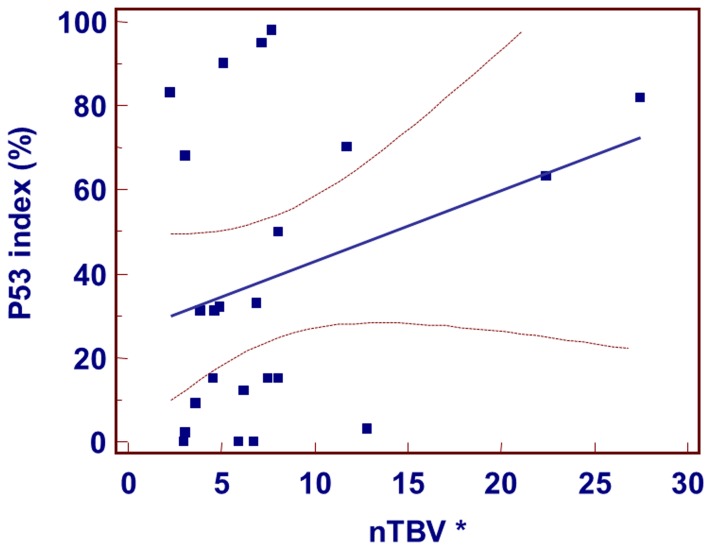
Scatter diagram and regression line display the relationship between nTBV and p53 index. There was no significant correlation between p53 index and nTBV value. Note– *nTBV: normalized relative tumor blood volume.

The Ki-67 labeling index also presented a strong positive correlation with nTBV parameters (r = 0.483, p = .014) (Figure 5). However, p53 expression status (r = 0.299, p = .166) did not present a significant correlation with the nTBV values (Figure 6). In LOOCV test for evaluating Ki-67 labeling index with nTBV values, Pearson correlation coefficient was 0.38 (p = .06).

From the univariate analysis, we found that the genetic alterations associated with an increase in nTBV included EGFR overexpression with PTEN loss, a non-methylated MGMT promoter and the Ki-67 labeling index. Thus, we performed a multivariable stepwise logistic regression analysis using the genetic alterations, which revealed that the Ki-67 labeling index was most strongly correlated with nTBV (p = .014) values.

**Figure 7:**
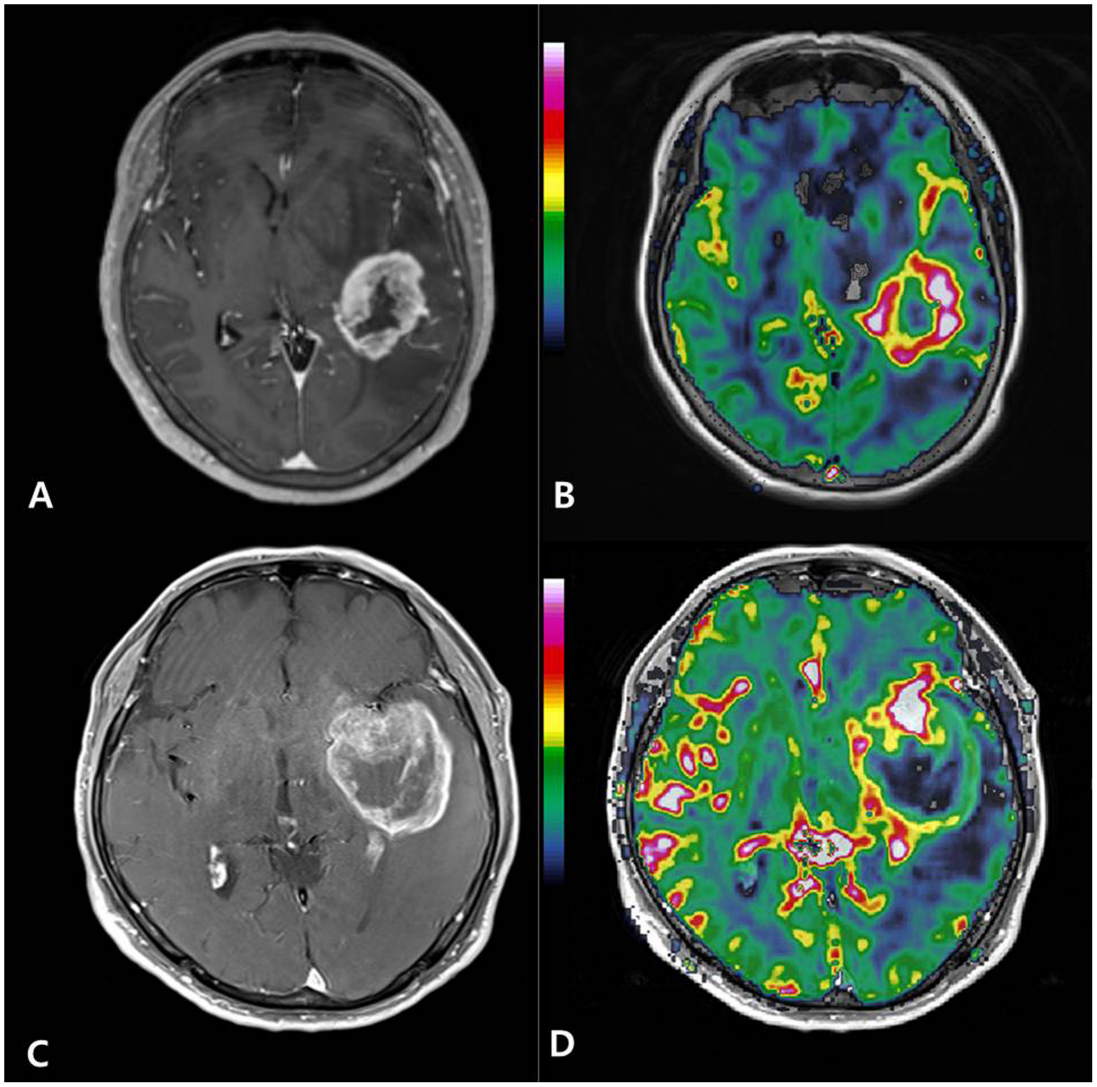
Glioblastomas with aggressive genetic profiles show high normalized relative tumor blood volume (nTBV). (A, B) A 66-year-old woman had glioblastoma with EGFR expression (3+), PTEN loss (+), MGMT methylation (−), and a Ki-67 index of 39%. The tumor showed high nTBV (27.5).(C, D) A 70-year-old man had glioblastoma with EGFR expression (3+), PTEN loss (+), MGMT methylation (−), and a Ki-67 index of 27%. The tumor showed high nTBV (12.84). (A, C) Contrast-enhanced T1-weighted axial image, (B, D) normalized relative cerebral blood volume (nCBV) map overlaid on structural contrast-enhanced T1-weighted axial image.

**Figure 8:**
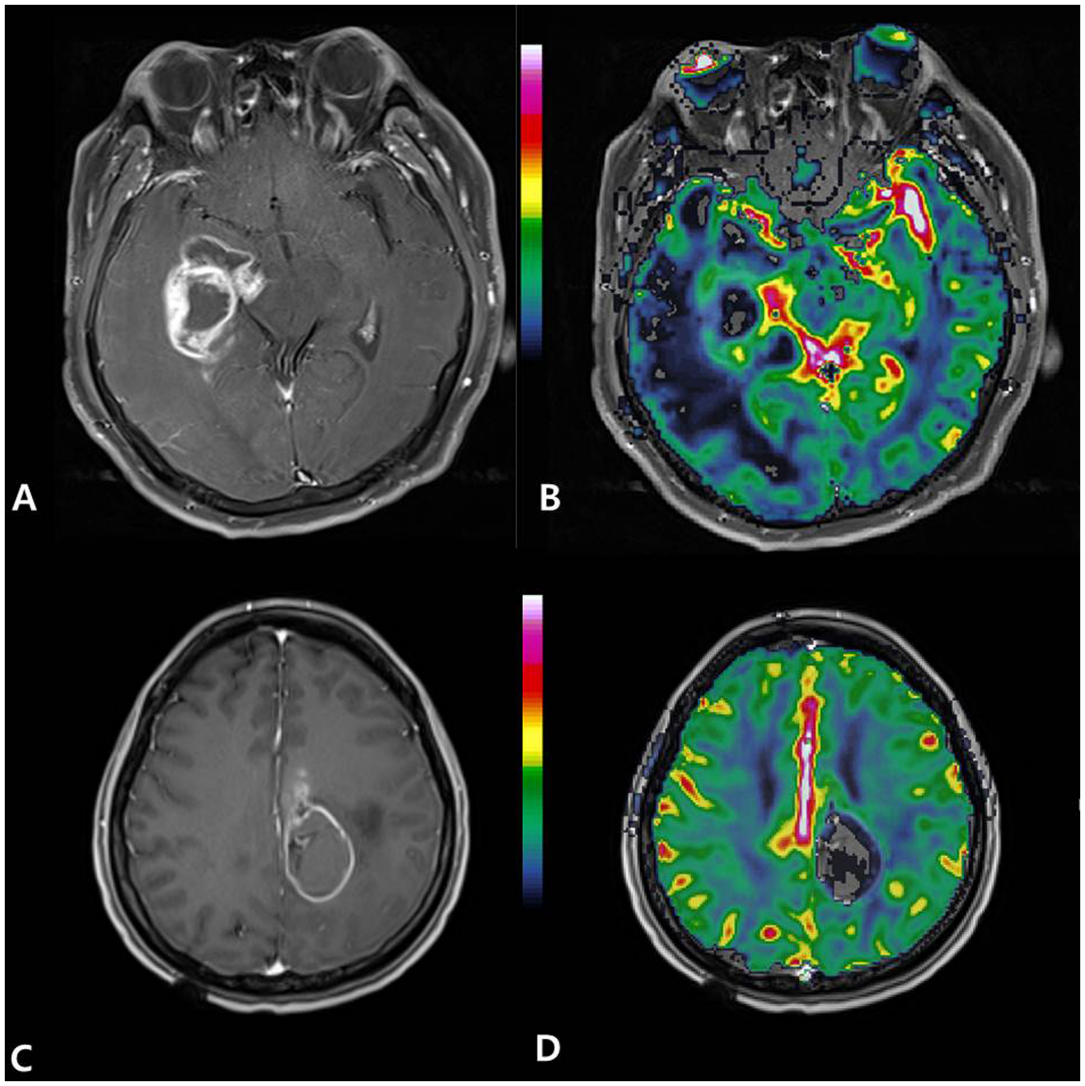
Glioblastomas with favorable genetic profiles show low normalized relative tumor blood volume (nTBV). (A, B) A 67-year-old man had glioblastoma with EGFR expression (2+), PTEN loss (−), MGMT methylation (+), and a Ki-67 index of 5%. The tumor showed low nTBV (4.3). (C, D) A 36-year-old woman had glioblastoma with EGFR expression (+), PTEN loss (−), MGMT methylation (+), and a Ki-67 index of 9%. The tumor showed low nTBV (4.59) (A, C) Contrast-enhanced T1-weighted axial image, (B, D) normalized relative cerebral blood volume (nCBV) map overlaid on structural contrast-enhanced T1-weighted axial image.

Figure 7 and 8 present the representative cases with high and low nTBV values, respectively, in which the genetic profiles are correlated with nTBV values.

## Discussion

In this study, we analyzed DSC-enhanced perfusion MR imaging of GBMs, which was correlated with genetic alterations of the tumors. We found that the EGFR expression-positive GBMs with loss of PTEN expression showed significantly higher nTBV values than those without loss of PTEN expression, and GBMs without MGMT promoter methylation also showed significantly higher nTBV values than those with MGMT promoter methylation. In addition, the Ki-67 labeling index had a positive correlation with nTBV parameter. Interestingly, the Ki-67 index was most strongly correlated with nTBV in GBMs. These genetic alterations have been known to be poor prognostic factors in GBMs, and the increase in nTBV is also a well-known predictor for progression in GBMs. Finally, we demonstrated that nTBV values were able to reflect some genetic alterations related to GBM prognosis.

EGFR is the most frequently amplified gene and a primary contributor to tumor initiation and progression in GBM [5]. EGFR amplification is associated with EGFR overexpression. The increased activity of EGFR promotes tumor growth through many different mechanisms, promoting survival, invasion, and angiogenesis. Many previous studies have demonstrated that EGFR stimulation activates many downstream signal pathways, including the phosphoinositide-3-kinase (PI3K)/Akt pathway. The EGFR becomes activated upon binding to epidermal growth factor (EGF) and recruits PI3K to the cell membrane. PI3K converts phosphatidylinositol-4,5-bisphosphate (PIP2) to the second-messenger molecule phosphatidylinositol-3,4,5-triphosphate (PIP3), which activates Akt. Activated Akt, in turn, activates the mammalian target of rapamycin (mTOR), which helps induce cellular proliferation and block apoptosis. Here is the point where PTEN plays an important role in inhibiting tumor proliferation. PTEN terminates the PIP3 signal that results in blocking the downstream pathways of EGFR-mediated cellular proliferation [5], [22], [23]. Therefore, GBMs with EGFR expression combined with PTEN gene loss present poor prognosis, and this may influence responses to EGFR-targeted therapy. The increased EGFR signaling by amplified EGFR expression with PTEN loss promotes increased angiogenesis, and increased TBV is a measure of microvasculature density and has previously been shown to reflect an underlying angiogenetic mechanism [24]; thus, tumors with EGFR overexpression with PTEN loss can have increased TBV.

MGMT is a repair protein that specifically removes promutagenic alkyl groups from the O6 position of guanine in DNA, thereby protecting tumor cells against alkylating agents [25], [26]. Loss of MGMT expression may be caused by the methylation of promoter CpG islands. MGMT promoter methylation is frequently present in GBM (45–75%) and has been associated with longer survival of GBM patients treated with temozolomide [27]–[31]. Therefore, the evaluation of the methylation status of the MGMT promoter is important for selecting treatment options.

Mutations in p53, a famous tumor suppressor gene, are found in a diverse variety of human tumors [32]. However, in this study there was no significant correlation with nTBV value.

Tumor cellularity and proliferation index have been shown to be associated with patient prognosis and survival [33], [34]. Ki-67 is a well-known biomarker representing the proliferative activity of tumor cells. An increase in the Ki-67 proliferation index has been found to be related with unfavorable prognosis in high-grade gliomas [33], [35], [36]. Considering that increased proliferation also means increased angiogenesis, an increased proliferation index may lead to increased TBV.

Rising interest and the need for non- or less-invasive techniques for the identification of the genetic alterations mentioned above in GBM, accompanied with exponential growth in the power and utility of imaging techniques such as MRI over the past 20 years, has led radiologists to search for imaging features or biomarkers reflecting genetic profiles of GBM. There have been several studies correlating various imaging features such as tumor border, enhancement pattern, peritumoral edema, multiplicity, T2 signal and intratumoral cystic change with genetic profiles of GBM, including EGFR and MGMT expression [4], [6], [37]–[39]. Recently, there has been increased interest in the EGFR status of GBM and EGFR-targeted therapy, including EGFRvIII (a mutated variant of EGFR with continuous autoactivation without EGF found frequently (20–30%) in GBMs) targeted therapy, which has been the subject of several studies relating imaging and EGFR status, especially using perfusion MR images [13], [20]. A very recent study revealed that EGFRvIII-expressing GBMs presented significantly higher relative TBV compared with those tumors lacking EGFRvIII expression [3]. When employing several promising therapies directed specifically at EGFRvIII, including EGFRvIII peptide vaccination and EGFRvIII-directed monoclonal antibodies, that have emerged recently, imaging-based identification of EGFR status becomes increasingly important [40], [41]. However, studies correlating perfusion MR imaging with other genetic alterations in GBM, including the genetic profiles analyzed in this study, have not yet been conducted.

Aside from the intrinsic limits of any retrospective study, several other limitations of this study should be mentioned. First of all, the sample size was rather small to generalize our findings and some results showed subtle statistical significance. Furthermore, LOOCV test demonstrated only borderline significance (p = .06) for the positive correlation between Ki-67 labeling index and nTBV, even though the p-value was 0.01 for their correlation on Pearson correlation analysis. The result may also be attributed to our small sample size. Thus further investigations with larger populations and more precise analysis such as histogram analysis are warranted to strengthen the statistical power. Secondly, we did not evaluate EGFRvIII and vascular endothelial growth factor (VEGF) expression status which are also known to be related to rTBV [24], as currently there are no available IHC methods and cytokine analysis (reverse transcription polymerase chain reaction, RT-PCR) for such analyses. Therefore, we could not assess the relationship between the perfusion parameters and EGFRvIII or VEGF expression status along with PTEN status. Lastly, the Nordic TumorEX software enabled only calculation of normalized rCBV values, other perfusion parameters such as cerebral blood flow could not be evaluated in this study.

Despite all these limitations, our study revealed that GBMs with aggressive genetic alterations tended to have high relative TBV values. These results indicate the potential of DSC-enhanced perfusion MR imaging as a noninvasive radiophenotypic biomarker for genetic profiles of GBMs that are crucial in predicting the prognosis and response to specific treatment and selecting a tailored therapy for GBM patients. Although pathologic analysis is still the gold standard in confirming genetic profiles, DSC-enhanced perfusion MR imaging, which may serve as a relatively non-invasive and convenient mean to predict the genetic profiles, could be a potentially useful alternative to surgery, especially for patients who cannot undergo invasive procedures. Furthermore, given that MR imaging is widely used in current clinical practice, our study could be a cornerstone of further research focused on determining the imaging biomarkers for various tumors, in addition to GBMs.

In conclusion, our results suggest that DSC-enhanced perfusion MR imaging could be a potentially useful alternative to surgery for prediction of the genetic profiles, thus serving as a guide to appropriate treatment selection, especially for patients who cannot undergo invasive procedures.
